# Lasting Impact: Exploring the Brain Mechanisms that Link Traumatic Brain Injury to Parkinson’s Disease

**DOI:** 10.1007/s12035-025-04706-x

**Published:** 2025-02-01

**Authors:** Samantha Edwards, Frances Corrigan, Lyndsey Collins-Praino

**Affiliations:** 1 Cognition, Ageing and Neurodegenerative Disease Laboratory, School of Biomedicine, The University of Adelaide, Adelaide, SA 5005 Australia; 2 Head Injury Lab, School of Biomedicine, The University of Adelaide, Adelaide, SA 5005 Australia

**Keywords:** Parkinson’s Disease, Traumatic Brain Injury, Neurodegeneration, Dopamine, Noradrenaline, α-synuclein

## Abstract

Development of Parkinson’s Disease (PD) is linked with a history of traumatic brain injury (TBI), although the mechanisms driving this remain unclear. Of note, many key parallels have been identified between the pathologies of PD and TBI; in particular, PD is characterised by loss of dopaminergic neurons from the substantia nigra (SN), accompanied by broader changes to dopaminergic signalling, disruption of the Locus Coeruleus (LC) and noradrenergic system, and accumulation of aggregated α-synuclein in Lewy Bodies, which spreads in a stereotypical pattern throughout the brain. Widespread disruptions to the dopaminergic and noradrenergic systems, including progressive neuronal loss from the SN and LC, have been observed acutely following injury, some of which have also been identified chronically in TBI patients and preclinical models**.** Furthermore, changes to α-synuclein expression are also seen both acutely and chronically following injury throughout the brain, although detailed characterisation of these changes and spread of pathology is limited. In this review, we detail the current literature regarding dopaminergic and noradrenergic disruption and α-synuclein pathology following injury, with particular focus on how these changes may predispose individuals to prolonged pathology and progressive neurodegeneration, particularly the development of PD. While it is increasingly clear that TBI is a key risk factor for the development of PD, significant gaps remain in current understanding of neurodegenerative pathology following TBI, particularly chronic manifestations of injury.

## Background

### Parkinson’s Disease

Parkinson’s Disease (PD) is estimated to affect between 7 and 10 million individuals worldwide [1]. The highest prevalence of PD cases occur in older individuals, with 80% of cases occurring in individuals over 65 years old [1], highlighting the importance of age as the largest risk factor for PD development [2]. Significantly, PD prevalence is rapidly increasing, with cases projected to reach more than 17 million globally by 2040, due to a combination of factors including the ageing population, decreased rates of smoking and continued exposure to industrial by-products [3, 4].

PD has a significant impact on overall function and quality of life, both for affected individuals and their families [5], with an estimated 1.2 million Years Lived with Disability (YLD) [1] and ranking 10th for disease burden among neurological disorders in 2019, based on Disability Adjusted Life Years [6]. Importantly, patients’ ability to independently complete instrumental activities of daily living, particularly cognitively-demanding tasks, is significantly impaired in people with Parkinson’s Disease (PwP), even those who live independently in the community [7], and this further declines over time [8], placing significant stress on caregivers and the health system [5, 9]. The economic impact of PD is also significant, with an estimated total economic burden of $51.9 billion for the approximately 1 million individuals in the US with PD, and these costs are projected to increase to almost $80 billion by 2037 [9]. As such, development of effective intervention strategies is critical to alleviate the rapidly growing burden of disease on the healthcare system for improved patient quality of life and care, and to minimise future economic impact.

Pathologically, PD is characterised by loss of dopaminergic neurons from the Substantia Nigra (SN), as well as the development of Lewy Bodies (LB), abnormal accumulations of aggregated α-synuclein, in the cytoplasm of neurons. Aggregation and accumulation of other proteins, notably amyloid-β [10] and tau [11], is also observed in the brains of PwP, although generally to a lesser extent than α-synuclein. The SN is an important motor nucleus which projects to the striatum via the nigrostriatal pathway and provides critical modulatory dopaminergic input to the Basal Ganglia (BG) circuitry. Thus, degeneration of the SN leads to an imbalance of inhibition and excitation to the motor cortex and consequent development of the characteristic motor symptoms associated with PD [12]. As such, PD is characterised clinically by its cardinal motor signs, including resting tremor, bradykinesia, muscle rigidity and postural instability (for review, see Weintraub et al. 2008 [13]). However, neuronal loss is not limited to these motor regions or exclusively to dopaminergic neurons, with noradrenergic neuronal involvement observed in early stages of disease and more general neuronal involvement at later stages [14]. Consequently, beyond its cardinal motor symptoms, PD is also associated with several non-motor symptoms affecting diverse body systems, including, but not limited to, autonomic dysfunction, mood disorders, such as depression and anxiety, and cognitive impairment (for review, see Schapira et al. 2017 [15]). In fact, among people who survive to 20-years post-diagnosis, over 80% develop dementia [16].

Additionally, in recent years it has been increasingly well-recognised that individuals display a constellation of symptoms in the decade(s) prior to diagnosis [17]. Collectively, these are known as prodromal symptoms and have been formally recognised by the International Parkinson and Movement Disorder Society in their formulation of criteria for prodromal PD, which unites symptom presentation with neuroimaging signs of abnormal tracer uptake in the dopaminergic system by single photon emission computed tomography (SPECT) or positron emission tomography (PET) [18, 19]. Non-motor signs include REM sleep behaviour disorder, olfactory dysfunction, autonomic disturbances (e.g. constipation, urinary dysfunction and erectile dysfunction), neuropsychiatric symptoms, particularly depression [18], and mild cognitive deficits [19]. Patients may also experience mild motor symptoms in the prodromal phase, reminiscent of, but less significant and widespread than, those observed in diagnosed disease, such as impaired balance, fine and gross motor slowing and gait changes [17, 18, 20].

Understanding of PD pathophysiology has developed significantly in the last 3 decades, although further work is still required in this area. Evidence of various pathological contributors, including inflammation [21], mitochondrial dysfunction [22], oxidative stress [23, 24], excitotoxicity [25], calcium dysregulation [26], apoptosis [27] and alterations to cellular proteostasis [28], iron homeostasis [29] and ferroptosis [30] have been identified in the brains of individuals with PD, suggesting a complex biological cascade, rather than a single mechanism or pathway, in the pathogenesis of PD. Indeed, various genetic, lifestyle and environmental factors have been associated with disease development and progression (for review, see Dunn et al. 2019 [31]). Environmental exposures, such as pesticides [32], heavy metals [33, 34] and head trauma [35], are of particular interest, as they provide potential opportunities for early detection and intervention. Recently, growing evidence and awareness of the link between traumatic brain injury (TBI) and PD has brought this to the forefront of current research efforts.

### Traumatic Brain Injury

The yearly incidence of TBI has been estimated at up to 69 million cases per year [36], according to a 2019 systematic review and meta-analysis with significant extrapolation, although the most recent Global Burden of Disease studies suggests a much lower figure, reporting approximately 27 million incident cases of TBI in 2016 and 2019 [37, 38]. The true incidence is likely somewhere between these figures; however, estimating this is difficult due to significant under-reporting, particularly of mild cases of TBI [36, 37]. Regardless, it remains that TBI is a major contributor to global injury burden, and has a significant impact on quality of life, with an estimated 6–10 million YLDs due to TBI globally each year [37, 38]. The main causes of TBI include falls, pedestrian and motor vehicle road injuries, sporting injuries, conflict and terrorism [36, 38]. Injury severity is primarily classified based on the Glasgow Coma Scale (GCS), duration of amnesia following injury and duration of loss of consciousness (LOC). Mild TBI, often known as concussion, accounts for the majority (75–85%) of cases and is generally defined as injury causing LOC for less than 30 min, amnesia for up to 24 h, and a GCS of 13–15. Moderate to severe TBI is defined as LOC for at least 30 min, amnesia for more than 24 h post-injury, and can be divided into moderate (approximately 11% of cases) and severe (approximately 8% of cases) presentation based on GCS, with a score of 9–12 representing moderate injury and a score of less than 9 representing severe injury [36, 39].

In addition to loss of consciousness and memory immediately following injury, TBI patients typically experience symptoms such as headaches, nausea, fatigue, dizziness and emotional issues in the days following injury [40]. Furthermore, motor dysfunction is common in the hours and days following injury, ranging from mild impairments in balance and coordination after mild injury [41–43] to more significant paresis and ataxia after more severe injury [44]. Decreased cognitive function [42, 43, 45], sleep disturbance [40], instances of neuropsychiatric dysfunction, particularly altered anxiety levels [46], and olfactory dysfunction are also seen to varying degrees following injury [47, 48]. In mild TBI patients, impairments generally resolve within weeks of injury, although a subset (~ 15%) of individuals experience persistent symptoms, many of which are also observed in PwP, including cognitive, neuropsychiatric [49] and motor dysfunction [50], hyposmia [51] and poor sleep quality [52]. Evaluating functional outcomes in the acute phase following moderate-severe injury is challenging due to prolonged loss of consciousness and hospital stays; however, preclinical studies indicate neurological, motor [50] and cognitive [53, 54] deficits in the hours and days following injury. Chronic outcomes, on the other hand, have been relatively well characterised, particularly cognitive dysfunction, with findings of cognitive impairment persisting for years following moderate-severe injury [49, 55]. Reports of motor deficits beyond a few years post-injury are limited and vary greatly in their findings; however findings indicate only minor chronic deficits, largely in the domains of balance and gait [50]. Furthermore, chronic neuropsychiatric complaints, particularly high levels of depression, as well as olfactory dysfunction, have also been observed in the years following moderate-severe injury, as have the development of sleep disturbances, dysphasia and bladder and bowel dysfunction [52, 56, 57].

**Fig. 1:**
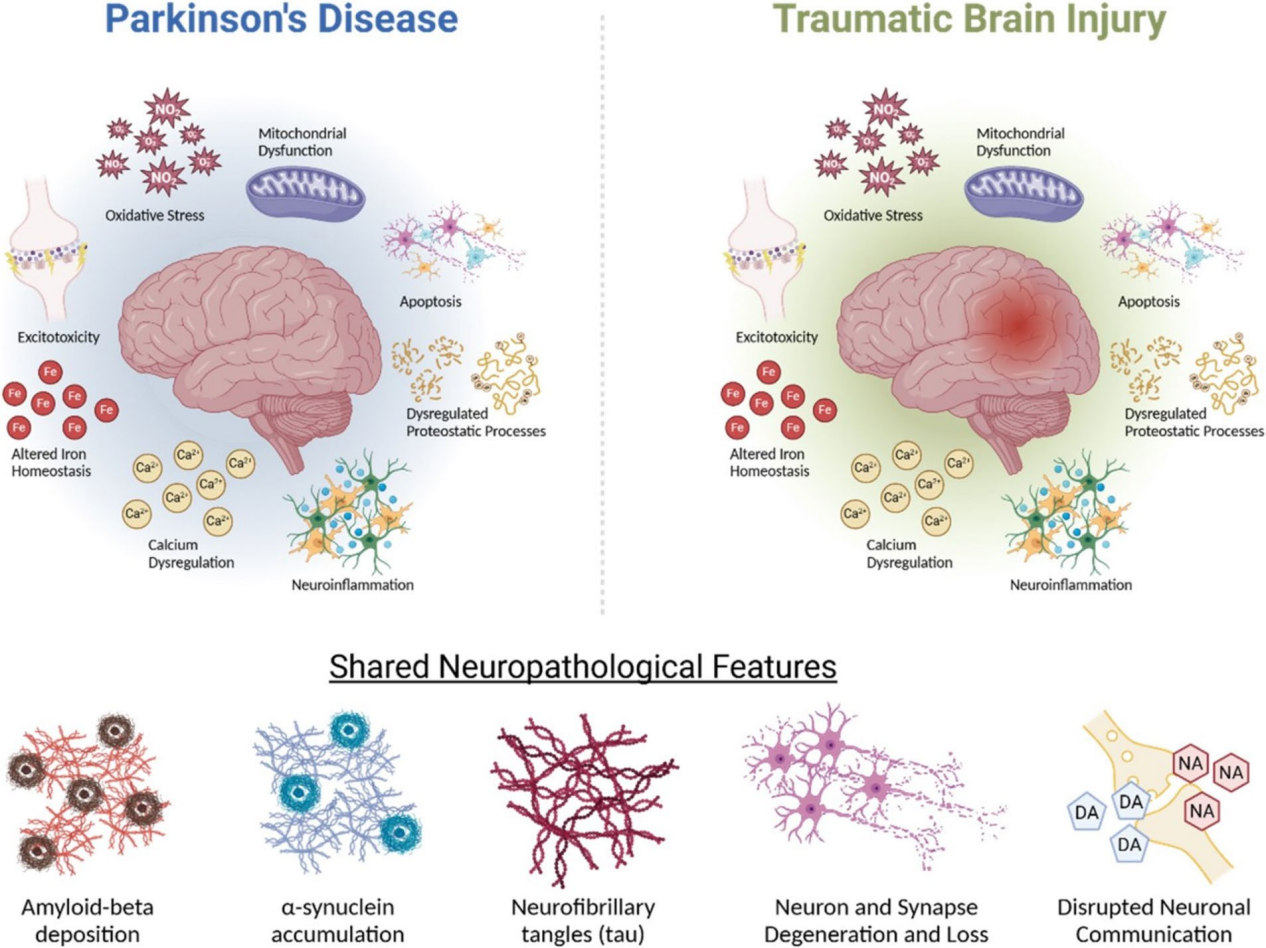
Shared pathophysiological features between Parkinson's Disease and the secondary injury cascade following traumatic brain injury. (Figure created with BioRender.com)

Notably, the primary mechanical insult of a TBI initiates ongoing secondary pathological cascades within the brain (for review, see Khatri et al. 2021 [58]). Elements of this secondary injury cascade overlap substantially with those relevant to the pathophysiology of PD (Fig. 1), including, but not limited to, the induction of neuroinflammation [59] and oxidative stress (with concomitant decreases in antioxidant capacity) [59, 60], excitotoxicity [61], mitochondrial dysfunction [62], calcium dysregulation [63, 64], altered brain iron homeostasis [65] and dysregulated proteostatic processes (such as the heat shock response, the unfolded protein response, the ubiquitin proteosome system and autophagy), leading to abnormal protein aggregation and apoptosis (for review, see Barker et al. 2023 [66]). These processes begin within minutes and have been observed in the hours and days following injury, with evidence that some may persist for years, or even decades, after the initial injury [67]. Furthermore, evidence of the neuropathological features observed in PwP, including dopaminergic and noradrenergic dysregulation and neuronal loss (eg [68–70]), as well as α-synuclein, amyloid-β and tau pathology (eg [71, 72]), have also been identified at various time-points following injury in humans and animal models (Fig. 1).

While the link between TBI and the later development of PD is widely accepted, and key parallels have been identified between the two pathologies, the biological mechanisms underlying this link remain poorly understood. In particular, TBI is generally regarded as a relatively acute phenomenon, with immediate pathophysiological features and functional changes that are well established in the literature; conversely, PD is an inherently chronic condition that takes years to develop and progressively worsens over time. As such, long-term manifestations of injury, and how these acute changes may evolve into chronic neurodegenerative pathology, remain poorly understood. *Importantly, increasing understanding of how neurodegeneration develops and progresses following injury is key to early detection and intervention for effective treatment.* Thus, this review will outline current understanding of both acute and chronic changes following TBI, with a focus on those related to the hallmark pathological features of PD: changes to both the dopaminergic and noradrenergic systems and α-synuclein aggregation. Importantly, we will also discuss existing evidence of how acute changes may lead to chronic pathology, and outline current gaps in our understanding of how TBI manifests chronically.

## Epidemiological Association Between TBI and PD

### The Link Between TBI and PD in the General Population

A causative link between head trauma and the later development of PD was first hypothesised by James Parkinson in his initial description of the disease in 1817 [73]. Despite this, there has historically been debate in the field, with many (e.g. [74–77]), but not all (e.g. [78–80]), early epidemiological studies supporting a link between a history of head injury and PD. These mixed findings were likely due, in large part, to recall bias and other confounding risk factors (see Wong et al., 2013 [81] for review). Nevertheless, a meta-analysis of 22 studies published between 1966 and 2012 found that the risk of developing PD was higher in patients who had previously had a head trauma of any severity [82]. Specifically, the Odds Ratio (OR) for PD development in head trauma patients was 1.57 (95% Confidence Interval (CI) = 1.35–1.83), indicating a 57% increase in risk in these patients, compared with the general population. Similarly, a more recent meta-analysis encompassing studies published between 1991 and 2021 reported an increased risk of PD (OR = 1.50; 95% CI = 1.23–1.83) following any type or severity of head injury [83]. However, variability in injury severity and the time between injury and PD diagnosis must also be considered when interpreting these results [81, 84]*.* One such meta-analysis, looking specifically at the association between mild TBI and development of PD at least 1-year post-injury, still found an association between injury and PD risk (OR = 1.45; 95% CI = 1.18–1.78) [85], suggesting that even less severe trauma can have a detrimental effect on long-term brain health. While the magnitude of this increased risk may appear modest, it is important to note that it is similar to that reported for other risk factors known to have a strong association with PD. For example, the OR for pesticide exposure and PD is 1.94 [86].

Further, in recent years, a growing body of research has utilised study designs aimed at mitigating issues associated with earlier work in this area, such as recall bias or reverse causation. Indeed, reverse causation has been raised as a potential confound by several earlier studies (e.g. [87]), with 60% of PwP reporting at least one fall (and 40% reporting recurrent falls), compared to just 15% of older adults in the general population [88]). This extends to the prodromal period, where a large epidemiological study by Camacho-Soto and colleagues (2017) [89] utilising US Medicare claims data from 89,790 individuals with PD and 118,095 comparable controls aged 65 years or older found the risk of TBI was greater in PD patients in the prodromal period, with risk increasing closer to time of diagnosis (5-years pre-diagnosis HR = 1.64; 95% CI = 1.52–1.77 vs. 1-year pre-diagnosis HR = 3.93; 95% CI = 3.74–4.13). To counter this, two notable prospective studies have been conducted in this area, with similar findings from both. Paul Crane and colleagues [90] analysed data from 7130 participants across three large prospective longitudinal studies of aging (the Religious Orders Study, Memory and Aging Project and Adult Changes in Thought study), of whom 865 had a self-reported history of TBI with LOC. TBI with LOC > 1 h, occurring at least 1 year before PD diagnosis, was strongly associated with both increased risk of PD diagnosis (OR = 3.56; 95% CI = 1.52–8.28) and symptom progression (OR = 2.23; 95% CI = 1.16–4.29). Conversely, no association was found with TBI causing LOC < 1 h, casting doubt over whether mild injury is indeed sufficient to cause PD [90]. Similarly, a 2015 study of 165,799 individuals aged 55 or older, who presented to inpatient/emergency department settings in California with either a TBI (n = 52,393) or a non-TBI trauma, such as a fractured bone (n = 113,406), reported a 44% increased risk of developing PD over a 5-to-7-year follow-up period in those with a history of TBI compared to those without [91]. This risk was present regardless of the nature of the injury (falls vs. non-falls), reducing the likelihood of reverse causation in these cases [91]. Furthermore, the authors found that both more severe and more frequent TBI doubled the risk of PD compared to mild or single TBI, and that TBI patients were diagnosed slightly earlier than non-TBI patients [91], suggesting a dose-dependent and cumulative effect.

Of note, TBI has also been shown to be associated with PD symptom development and progression. Significantly, Crane et al. reported progression of parkinsonian signs, assessed with a modified version of the Unified Parkinson’s Disease Rating Scale, to be moderately associated with TBI (OR = 1.75; 95% CI = 1.33–2.29) [90]. Furthermore, in a retrospective case–control study of veterans with PD, those with a history of PD had more (although not statistically significant; p = 0.06) documented motor symptoms [92]. Similarly, a prior history of TBI can have implications for those with PD beyond affecting motor symptom progression alone [90]. In a study of 25 individuals with PD with a history of mild-moderate TBI and 25 demographically matched controls without such a history, the PD-TBI group showed greater declines on the Mattis Dementia Rating Scale, particularly in executive function, over a two-year follow-up period compared to the group without a history of TBI [93]. Similarly, Joyce et al. (2020) reported in a cross-sectional study that a history of TBI was associated with decreased scores of global cognition, executive function, memory and language, as well as increased depressive symptoms, in those with PD [94].

### The Link Between PD and TBI in Military Veterans

Among military veterans, in particular, there is a significant amount of research establishing an increased risk of developing PD. This is perhaps not surprising, given that many of those in active military service experience a TBI. For example, among US military veterans deployed in Iraq and Afghanistan post-09/11, it is estimated that between 9 and 28% of soldiers experienced a TBI [95]. The majority (~ 75%) of these injuries were mild in nature, similar to a concussion, with long-term exposure to explosive weapons cited as the most significant cause of brain injury [96]. This type of injury, often termed blast TBI, differs from TBI sustained as a result of falls, collisions or other mechanisms of direct trauma. While injury in blast TBI results from shockwave propagation and pressure changes, its associated pathology largely parallels that seen following TBI induced by direct trauma. Despite these similarities, blast TBI is also associated with distinct outcomes. Importantly, in addition to axon shearing and axonal injury, blast TBI causes damage to vasculature and the blood–brain barrier and changes in intracranial pressure, as well as more widespread effects on various organ systems and the development of systemic inflammation (reviewed in Kim et al., 2023 [97]). Importantly, both blast and non-blast TBI can have ramifications long beyond the initial injury alone. In line with this, Gardner and colleagues (2017) [98] carried out a cross-sectional cohort study of motor function in retired military veterans aged 50 + with (n = 78) and without (n = 85) a prior history of TBI, all without a diagnosis of PD. Those with a history of moderate-severe TBI were more likely to have reported a fall in the past year (RR = 2.5; 95% CI = 1.1–5.4), had higher scores of global motor dysfunction and higher scores of postural and gait instability (although not higher scores for tremor, muscle rigidity or bradykinesia). Interestingly, similar impairments were not noted in those with a history of mild injury. This contrasts, however, with the results of a follow-up retrospective cohort study in 325,870 individuals, half with a prior history of TBI, by the same group using the US Veterans Health Administration database. Even after adjusting for demographics and medical/psychiatric comorbidities, mild TBI was associated with a 56% increased risk of PD, while more severe injury was associated with an 83% increased risk, in military veterans [35].

More recently, a large case control study looked at the association between early military-service related trauma and Parkinson’s disease in 71,933 US military veterans (and 287,732 matched controls). The study found that TBI, as well as PTSD, increased the odds of later PD at all preceding 5-year intervals, up to 60 years prior, with these two variables showing great synergy with each other [99]. This is in line with work by White and colleagues (2020), which also showed that PTSD can further increase the risk of PD in veterans with a history of TBI (a 2.69-fold and 3.70-fold excess relative risk of PD in veterans with mild and moderate-severe injury, respectively, when PTSD was present, versus a 2.17-fold and 2.80-fold excess risk in the absence of PTSD) [100]. This is significant, as there is known to be a high prevalence of PTSD among military veterans, compared to the general population, with a meta-analysis focused on veterans of Operation Enduring Freedom/Operation Iraqi Freedom reporting a PTSD prevalence of 23% [101]. This raises additional concerns about the need for continued monitoring of veterans with a dual history of both TBI and PTSD. This is particularly significant when you consider that military veterans have been found to be three times as likely as a member of the general population to develop PD [102].

Taken together, the results of multiple studies in both the general population and military veterans strongly support the link between TBI and incident PD. Such a link is supported by pathological changes seen within the brain following TBI, with key parallels to the pathophysiology of PD.

## Biological Association between TBI and PD.

### Dopamine

Progressive loss of dopaminergic neurons within the SN, which exceeds that seen in healthy ageing, is one of the defining characteristics of PD [14, 103]. On average, at autopsy, approximately 48% loss of neurons was seen at the onset of symptoms (mean age 60 years) and 75% loss overall in the SN of individuals with PD (mean age 75 years), compared to a more modest 33% in ageing healthy controls (ages ranging from 20 to 90 years) [103]. As described above, degeneration of these neurons is the key driver of characteristic motor symptoms associated with PD [12]. Motor symptom presentation and consequent clinical diagnosis of PD typically does not occur until at least 50% of dopaminergic SN neurons are lost [104, 105], and striatal dopamine deficiency exceeds 80% [106–108], presenting challenges for effective treatment at this late stage. As such, improved understanding of causative factors and disease processes is critical for better prevention and treatment of disease progression in the early stages.

#### Clinical Evidence of Dopaminergic Dysfunction following TBI

**Fig. 2:**
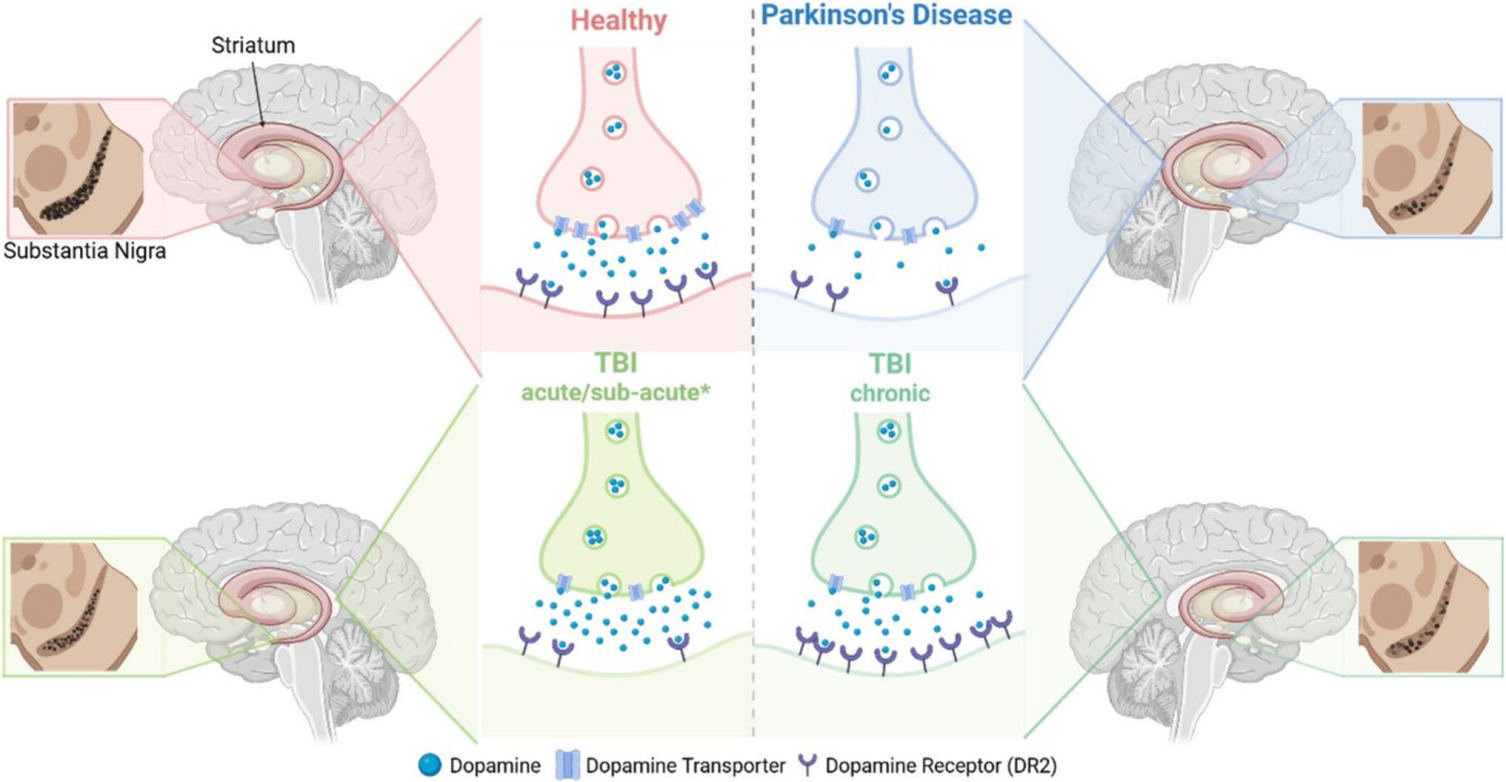
The constellation of changes seen within the dopaminergic system in healthy individuals, PwP and TBI at acute/sub-acute and chronic time-points. Significant dopaminergic neuronal loss is observed in the SN in PwP, similar to, but greater than, that seen post-TBI. Within the striatum, dopamine expression is reduced in PD, increased acutely following TBI and relatively unchanged chronically; DaT expression is reduced in PD and TBI, regardless of time post-injury; and DR2 expression is reduced in PD and acutely following injury, but increased chronically. Acute = hours-days post-injury; Sub-acute = days-weeks post-injury; Chronic = weeks-years post-injury. *The majority of findings of dopaminergic changes acutely following injury have only been observed in pre-clinical models, and are thus speculated as proposed changes and have not yet been verified in clinical cohorts. (Figure created with BioRender.com)

Clinically, volumetric MRI also supports a reduction in the volume of the SN and striatum post-injury, taking at least six months to become evident, and persisting to 30 years post-injury [68]. Reductions in the dopamine transporter (DaT) in the striatum have also been reported with single-photon emission tomography (SPET) within the first year following moderate-severe injury [109–111]. However, these changes in DaT differ to those with PD, as PD preferentially causes reductions in the putamen compared to the caudate, with greater asymmetry compared to that seen post-TBI [111]. Dopamine receptor changes have also been observed via SPET, with reductions in DR2 noted in the striatum in a small cohort of TBI patients an average of 4–5 months post-injury [110], but a contrasting modest increase in DR2 expression seen at one year post-injury [109], suggesting that receptor expression may evolve over time post-TBI. This too is in contrast to changes seen in PwP, where striatal DR2 is higher than controls in early disease, but similar to or lower than controls approximately 4 years after symptom onset (Fig. 2) [112]. Overall, given the small cohorts and large variation in time post-injury (60 days—30 years), it is impossible to speculate about the exact time course of nigrostriatal dopamine alterations post-injury. Furthermore, changes to the dopaminergic system immediately following injury remain largely unexplored in these clinical cohorts, limiting our understanding of pathology occurring within the dopaminergic system acutely following TBI.

#### Preclinical Evidence of Dopaminergic Dysfunction following TBI

A growing body of evidence from preclinical studies supports that there is dopaminergic dysfunction, including loss of dopamine neurons, changes in dopamine expression and alterations in pathways related to dopamine transmission, reuptake and metabolism, particularly acutely, following TBI. To date, however, the investigation of changes within the dopaminergic system more long-term following injury remains a noticeable gap in the literature. In the follow subsections, we discuss preclinical evidence to date about dopaminergic dysfunction following TBI, and highlight areas for future research.

#### Neuronal Loss

Preclinical animal models may help to address at least some of these limitations, particularly in the examination of acute dopaminergic changes post-injury. A progressive loss of dopaminergic neurons from the SN over time, as assessed via tyrosine hydroxylase (TH) staining, is seen, increasing from 15% loss at 11 days following lateral fluid percussion (LFP) injury to a 25–30% loss seen at 1–6 months following injury in models of LFP injury [69], midline fluid percussion [113] and Controlled Cortical Impact (CCI) [114] (Fig. 2). More chronically, however, in work from our own group, TH levels were unchanged in the SN at 12-months following diffuse mild, repetitive mild and moderate-severe TBI induced using the weight drop acceleration model, compared with shams [115]. However, this was only measured by western blot analysis, and therefore whether neuronal loss was present at this time-point following injury is unclear.

While it appears that the magnitude of dopaminergic neuronal loss following injury is not as severe as that seen in PD patients (up to 75%), even at delayed time-points, it is clear that injury can disrupt the dopaminergic system through neuronal degeneration and death. This is significant, as such disruption may create an underlying vulnerability in the dopaminergic system that can be exacerbated by other risk factors, increasing the likelihood of PD development. In line with this, administration of the pesticide paraquat (10 mg/kg IP) at either 3- or 6-days following TBI accelerated dopaminergic neuron loss, such that the TBI + paraquat rats had double the dopaminergic loss at 11 days; however, by 26 weeks post-injury, the TBI alone and TBI + paraquat rats had similar levels of dopaminergic neuron loss (30%) [69]. Thus, synergistic effects may only be present immediately following injury, or the synergistic effect of paraquat may act to accelerate neurodegenerative processes following injury, rather than simply increasing overall PD risk. Alternatively, this model utilised a single administration of the pesticide, which is in contrast to long-term exposure patterns seen in clinical populations, suggesting that more long-standing exposure to pesticides or other pathological factors prior to or surrounding the time of injury may be required for a long-term synergistic effect to be observed on neurodegeneration and PD risk.

#### Dopamine Expression

Nigrostriatal connections from the SN provide the main source of dopamine input to the striatum, and therefore loss of these neurons following injury results in decreased dopaminergic transmission to the striatum. However, parallel to this dopaminergic neuronal loss in the SN, acute increases in dopamine expression, particularly in the striatum, have been observed following injury (Fig. 2). This has been suggested as a compensatory mechanism to increase dopamine levels, by increasing dopamine synthesis in the striatum itself, a phenomenon also observed in response to SN degeneration in PD [116]. For example, dopamine levels, measured directly by High Performance Liquid Chromatography, were increased in the contralateral frontal cortex and whole striatum at 1 h and in the ipsilateral cortex at 1 day following CCI injury [117], and TH phosphorylated at Ser40, which has been directly associated with dopamine synthesis, was increased in the striatum at 2 and 7 days after a blast TBI. However, Ser40-TH was decreased in the whole brain and SN at these time-points [118], likely representative of SN dopaminergic neuronal loss (as discussed above), and therefore decreased dopamine synthesis, in these regions.

Furthermore, dopamine levels returned to normal in the cortex and striatum by 7 days and were maintained at this level until 28 days post-CCI injury [117]. More chronically, work from our own group reported that TH levels measured using Western blot were also unchanged in the striatum and prefrontal cortex (PFC) at 12-months following diffuse mild, repetitive mild and moderate-severe TBI compared with shams [115]. Continued compensatory upregulation of dopamine release in response to progressive loss of dopaminergic neurons from the SN may explain this return to control levels; however, this likely does not represent a return to a physiologically normal state, as the primary source and transmission of dopamine remains depleted. While the results of these studies indicate that dopamine levels are affected by injury and alterations typically don’t persist long-term, investigation of changes to dopamine expression in particular regions of interest and over the full time-course post-injury is patchy. Importantly, more systematic analysis of dopamine changes, throughout the entire time-course post-injury in different models of injury, is required to understand the nuanced pathology occurring in these models and how this may relate to disease development.

#### Dopamine Transmission, Reuptake and Metabolism

Alongside dopaminergic neuronal loss post-TBI, alterations in dopamine transmission pathways have also been observed acutely following injury in preclinical models (Fig. 2). For example, no changes were observed in D2 dopamine receptor expression at either 14 days in the striatum following CCI injury [70], or to D1 dopamine receptor expression at 12-months post-injury in the striatum or SN, with the only change to TH at this time-point being an increase in the following mild diffuse, but not moderate-severe, injury compared with both shams and repetitive mild injury [115]. This is in contrast to clinical findings of a reduction in receptor expression sub-acutely and an increase in expression in the chronic phase post-injury in the striatum, with an early increase, followed by a modest decrease in expression, which is also seen in PwP. However, receptor expression following injury remains largely unexplored pre-clinically, with a limited selection of receptors in a limited array of injury models and time-points post-injury investigated to date.

Dopamine re-uptake tends to decrease following injury, potentially as an additional compensatory mechanism to increase dopamine levels in regions experiencing reduced dopaminergic transmission following injury. DaT expression has been found to be unchanged at 1-day post-injury and decreased in the ipsilateral cortex at 7 days [119], the striatum at 14 days [70], bilaterally in the cortex at 28 days [119] and in the SN at 30 days [120] following various models of CCI injury. Chronic changes to dopamine reuptake have not been investigated pre-clinically.

Dopamine metabolism, determined by the ratio of dopamine to DOPAC (a metabolite of dopamine), was increased in the striatum 1-h following CCI injury, which is in contrast to findings of increased dopamine levels at this acute time-point and may represent a direct pathological response to injury. However, this returned to normal within a day [117], and more chronically, levels of catechol-O-methyl transferase (COMT), a catecholamine-metabolising enzyme, were unchanged in the PFC, striatum and SN at 12-months following mild, repetitive mild and moderate-severe TBI [115]. However, acute changes to COMT activity in key dopaminergic regions, and the exact role of COMT in injury processes, requires further investigation.

These findings suggest a complex array of changes to dopamine transmission, reuptake and metabolism post-TBI (Table 1). As such, the impact of injury on dopamine transmission pathways, particularly the time course of changes post-injury, requires further investigation. Importantly, greater understanding of the pathological mechanisms occurring within the dopaminergic system following injury is crucial for elucidating implications of injury for the development of neurodegeneration later in life.

**Table 1:** Summary of dopaminergic changes following TBI

**Region**	**Model**	**Outcome**			**Reference**
		Acute	Sub-Acute	Chronic	
**Whole brain**	Mild blast TBI (rat)	↓Ser40	↓Ser40		Acosta et al., 2019
**Cortex**					
Dopamine^a^	CCI (rat)	↑	↔		Massucci et al., 2004
DaT	CCI (rat)	↔	↓		Yan et al., 2002
**Prefrontal Cortex**					
DA Synthesis^b^	Weight drop TBI (rat) (mTBI, rmTBI, msTBI)			↑	Wee et al., 2024
DR2				↔	
COMT				↔	
**Striatum**					
Volume	Human brain (MRI)		↓	↓	Jenkins et al., 2018
DaT	Human Brain (SPET)		↓	↓	Wagner et al., 2014 Donnemiller et al., 2000 Jenkins et al., 2020
DR2			↓		Donnemiller et al., 2000
				↑	Wagner et al., 2014
			↔		Wagner et al., 2005
	Weight drop TBI (rat) (mTBI, rmTBI, msTBI)			↔	Wee et al., 2024
Dopamine^a^	CCI (rat)	↑	↔		Massucci et al., 2004
DA Synthesis^b^	Mild blast TBI (rat)	↑Ser40	↑Ser40		Acosta et al., 2019
	Weight drop TBI (rat) (mTBI, rmTBI, msTBI)			↔	Wee et al., 2024
DaT	CCI (rat)		↓		Wagner et al., 2005
DA Metabolism	CCI (rat)	↑	↔		Massucci et al., 2004
COMT	Weight drop TBI (mTBI, rmTBI, msTBI)			↔	Wee et al., 2024
**Substantia nigra**					
Volume	Human Brain (MRI)		↓	↓	Jenkins et al., 2018
Dopaminergic neuronal loss	Mod. LFP injury (rat)		↓*	↓**	Hutson et al., 2011
	Mod. LFP injury + pesticide (rat)		↓**	↓**	
	Midline FP injury (rat)			↓**	van Bregt et al., 2012
	CCI (rat)			↓**	Acosta et al., 2015
DA Synthesis^b^	Mild blast TBI (rat)	↓Ser40	↓Ser40		Acosta et al., 2019
	Weight drop TBI (mTBI, rmTBI, msTBI)			↔	Wee et al., 2024
DR2				↔	
DaT	Mod. CCI (mouse)		↓		Impellizzeri et al., 2016
COMT	Weight drop TBI (mTBI, rmTBI, msTBI)			↔	Wee et al., 2024

*CCI* Controlled Cortical Impact injury, *COMT* catechol-O-methyl transferase, *DA* Dopamine, *DaT* Dopamine Transporter, *DR2* Dopamine Receptor 2, *LFP* Lateral Fluid Percussion injury *MRI* Magnetic Resonance Imaging; mTBI: mild TBI; msTBI: moderate-severe TBI; rmTBI: repetitive mild TBI; SPET: single-photon emission tomography

a: measured directly by High Performance Liquid Chromatography; b: Tyrosine Hydroxylase measured

↓* 15% loss of neurons ↓** 25–30% loss of neurons ↔ no change compared to control or baseline

Taken together, both preclinical and clinical findings suggest potentially widespread disruptions of the dopaminergic system following injury. While specific investigation of dopaminergic neuronal loss following injury is limited primarily to acute preclinical models [69, 113, 114]**,** clinical findings indicate reduced SN volume in the chronic phase [68] and neuronal loss has been shown to increase over time following injury, up to 26 weeks post-injury preclinically [69]. This suggests that while acute dopaminergic dysfunction appears to resolve following injury [115], these alterations or other pathological mechanisms may contribute to progressive neuronal injury and loss which could continue to worsen beyond the initial injury and persist chronically. However, further work investigating the chronic effects of injury on the dopaminergic system are required to better understand this, as well as how these changes relate to the emergence of PD in certain individuals.

### Noradrenaline

**Fig. 3:**
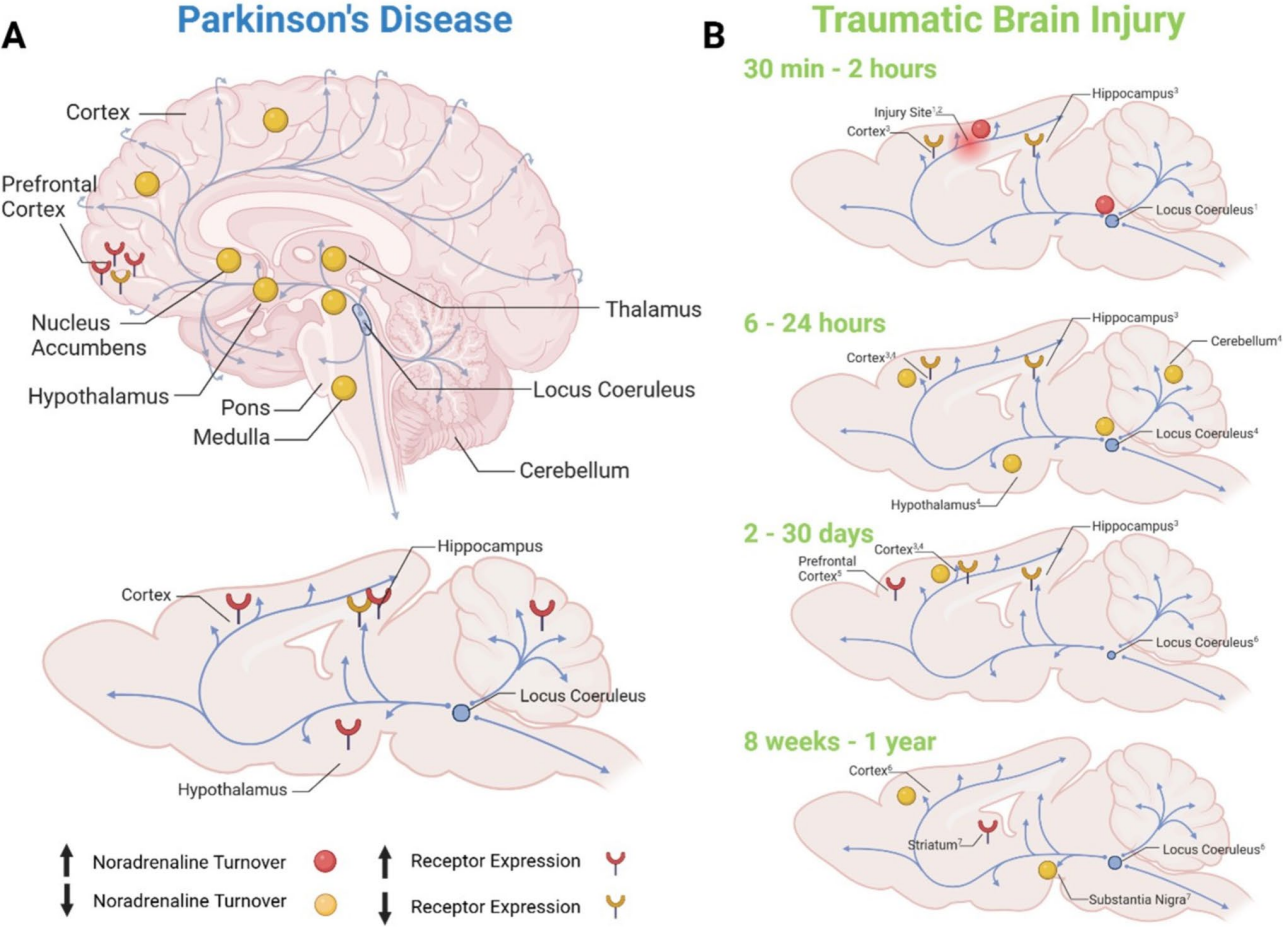
Noradrenaline turnover is decreased in several key noradrenergic regions in PwP (**A**). Similarly, in TBI, while turnover increases acutely around the injury site and in the main noradrenergic nucleus, the Locus Coeruleus (LC), it decreases in these regions, as well as those synaptically connected to the LC, more chronically (**B**); shown in preclinical models up to 1 year post-injury). Receptor expression is generally increased in PD. Following TBI, receptor expression tends to decrease in the hours and days following injury, but has shown increases in some regions more chronically (from 14 days). However, changes to receptor expression vary greatly depending on specific sub-types (α_1A_, α_1B_, α_1D_, α_A2A_, β_1_; not shown) and regions assessed, with the majority showing no change. Minimal work has been done investigating noradrenergic changes following injury in humans, with the main finding being acute increases in plasma noradrenaline levels (not shown). Blue arrows show noradrenergic pathways from the Locus Coeruleus in the human brain (**A**) and rodent brain (**B**). 1: Levin et al., 1995; 2: Dunn-Meynell et al., 1998; 3: Prasad et al., 1992; 4: Dunn-Meynell, 1994; 5: Kobori et al., 2011; 6: Fujinaka et al., 2003; 7: Wee et al., 2024;

While degeneration of dopaminergic neurons in the SN is recognised as the pathological hallmark of PD, the noradrenergic system has also been identified as playing a significant role in disease pathogenesis (for review, see Paredes-Rodriguez et al., 2020 [121]). Clinical studies of PwP and matched controls have demonstrated decreases in noradrenaline concentration and noradrenaline transporter density in key brain regions, including, but not limited to, the primary motor and sensory cortices, thalamus, hypothalamus, brainstem nuclei, nucleus accumbens and SN [122–125] (Fig. 3). The locus coeruleus (LC), in particular, is the main source of noradrenaline in the brain and plays an important role in cognitive function through extensive connections throughout the cortex [126, 127]. Critically, PwP display decreased MRI intensity and significant noradrenergic neuron loss in the LC at early stages of disease [128], which progressively worsens throughout the course of disease [129, 130], and declines to as little as 20% of control levels on autopsy [128]. This loss has been associated with the development of PD symptoms including RBD, depression, dysautonomia and cognitive dysfunction, and is therefore critically important in our understanding of disease development and progression [128, 130]*.* Furthermore, noradrenaline has been suggested to play a neuroprotective role in PD progression, with regions rich in noradrenaline, such as portions of the nucleus accumbens, relatively spared from dopamine loss [124].

#### Clinical Evidence of Noradrenergic Changes following TBI

Current understanding of noradrenergic changes following TBI is limited, and results vary significantly [131]. Post-mortem findings from the LC of patients with a history of multiple mild TBIs indicate neuronal loss in this important noradrenergic nucleus [132]; however, more detailed investigation into changes following single TBIs of varying severities and throughout a broader time-course post-injury are warranted. Interestingly, noradrenaline has been observed to be markedly elevated in the plasma acutely following injury [133, 134], generally suggested as a compensatory mechanism to increase cerebral and peripheral perfusion pressure post-injury. In line with this, treatment with noradrenaline is common for patients with severe traumatic brain injury, although debate persists regarding whether this is indeed beneficial for patients’ neurological outcome and mortality [135]. Indeed, higher noradrenaline levels at baseline (within 24-h of injury) have been associated with poorer prognosis, including mortality and functional outcomes assessed by the Glasgow Outcome Scale (GOS), at 6 and 12-months following injury [133, 134]. Furthermore, treatment with β adrenergic receptor blockade and alpha 2-adrenergic receptor agonists in the days following moderate-severe TBI requiring hospital admission has also been demonstrated to improve patient outcomes, including GCS and mortality, up to 6-months post-injury [136–138], likely through the reduction of cerebral oedema and improved glymphatic and lymphatic clearance of cellular debris. As such, initial increases in adrenaline may play a neuroprotective role by maintaining sufficient cerebral perfusion, although counteracting this beyond the early acute stages appears to be beneficial; thus, the wider ramifications of noradrenergic upregulation require further scrutiny. Furthermore, the role of noradrenaline elevation in the context of noradrenergic neuronal function and integrity remains unclear and, to date, no clinical studies have directly assessed noradrenaline levels in the brain or plasma beyond a few days post-injury. One study of severe TBI patients undergoing rehabilitation did, however, find decreased levels of plasma tyrosine, a precursor of noradrenaline, upon admission (approximately 1-month post-injury) and at discharge (approximately 4-months post-injury), which was suggested to equate to low brain tyrosine levels and therefore reduced noradrenaline synthesis [139]. These findings suggest that initial increases in noradrenaline do not persist long-term; however, further investigation of noradrenaline changes chronically following injury, and the relevance of this for the later development of neurodegeneration, is warranted.

#### Preclinical Evidence of Noradrenaline Changes following TBI

Evidence from preclinical studies demonstrate similar changes within the noradrenergic system, suggesting an acute increase in noradrenaline after injury (Fig. 3). Greater noradrenaline turnover has been reported in the first 30 min after somatosensory cortex contusion injury [140], and up to 2 h post-injury in the same model [141], particularly notable at the site of injury and closely surrounding regions. Interestingly, in this model, an increase in noradrenergic turnover was also observed bilaterally in the LC, distal from the site of injury, at 30 min post-injury, but only in response to impact on the left side of the brain, and not the right [140]. The significance of this discrepancy is unclear, but suggests that changes within the LC following injury are subtle and variable, and may not present uniformly in all cases. Upregulation in noradrenaline turnover appears to be transient, however, with no change compared to controls in the LC at 2 h after somatosensory cortex contusion injury [141], and decreases reported around the injury site and in the cortex, hypothalamus and cerebellum by 6 h following sensorimotor cortex contusion injury, which are maintained at 24-h and also observed in the LC at this time-point [142]. Overall, this pattern of changes to noradrenaline expression throughout the brain aligns with plasma changes observed in clinical populations, with an initial, potentially neuroprotective, increase [140, 141], followed by a decrease to baseline levels, and potentially lower, following the acute phase. This is important when considering management of TBI sequelae, with a relatively short window in which therapeutic increases in noradrenaline may be beneficial. Furthermore, these decreases in turnover are consistent with changes seen in the noradrenergic system in PwP [123], suggesting a potential key role for noradrenergic changes following injury in the development of neurodegenerative pathology.

More chronically, following diffuse weight-drop injury, noradrenaline turnover was unchanged in the cortex at 2 days post-injury, but decreased up to 8 weeks post-injury [143]. Concurrently, a decrease in the cross-sectional area of the LC stained with dopamine beta hydroxylase (DβH), an enzymatic catalyst of the conversion of dopamine to noradrenaline used as a proxy for noradrenaline expression, was decreased at 1 and 2 weeks post-injury, but returned to control levels at 4 and 8 weeks [143]. In work from our own group, DβH levels were decreased in the SN, but not the striatum or PFC, at 12-months following moderate-severe diffuse axonal injury (although not after either mild or repetitive mild injury) [115], suggesting that this region may be particularly sensitive to changes in noradrenergic function, which could potentially have implications for risk of PD development. However, neuronal integrity and neurotransmitter expression in the LC were not investigated in this chronic study, limiting our understanding of the mechanisms driving this decrease. Further investigation of noradrenergic expression in the LC and SN specifically, throughout a broad time-course post-injury, is required to better understand this aspect of the injury response and its role in neurodegeneration.

In contrast to the increase in noradrenergic turnover observed acutely following injury, noradrenergic receptor expression appears to be decreased (Fig. 3), although current research surrounding receptor changes following injury is limited. In the acute phase following injury, α_1_ adrenergic receptor density was decreased in the hippocampus and contralateral cortex within 30 min and up to 30 days following moderate LFP injury [144], contrasting with changes seen in PD, where α_1_ increased in the PFC of human post-mortem tissue and in the cortex, hippocampus and hypothalamus of the diseased rat brain [145, 146] (Fig. 3). However, α_1A_ adrenergic receptor mRNA was increased in the medial PFC at 14 days following CCI injury, although no changes were observed in the levels of either α_1B_ or α_1D_ mRNA expression [147].

To date, noradrenergic receptor expression in relevant regions for PD pathology, particularly the LC and SN, have not been characterised acutely, and findings regarding chronic changes are limited too. Recently, our own group demonstrated increases in α_2A_ adrenoceptors in the striatum (concomitant with DβH reductions in the SN) at 12-months following moderate-severe, but not mild or repetitive mild, diffuse axonal injury. However, these changes were not seen in the PFC or SN, and we observed no changes in α_1A_ or β_1_ adrenergic receptor expression in the PFC, striatum or SN at 12-months following any injury severity [115]. In contrast, increased α_1_ and β_1_ have been reported in the PFC of human PD post-mortem tissue and the diseased rat brain, while α_2_ is decreased or unchanged in both tissues [145, 146]. As such, there appears to be significant variability in specific receptor subtypes affected in different regions and at different time-points post-injury, and how these changes may contribute to the development of neurodegenerative pathology requires further characterisation. Furthermore, while receptor expression appears to follow different trends in acute TBI and PD, with only isolated regional and subtype-specific decreases seen in PD, chronic manifestations of injury more relevant to PD pathology remain largely unexplored.

Overall, findings from these studies suggest that TBI can cause both acute and longer-term alterations to the adrenergic system. While acute changes may be compensatory or neuroprotective, injury appears to cause longer term damage to the LC, resulting in downstream effects within the extensive noradrenergic networks extending from this nucleus, which may predispose these regions to further damage and neurodegeneration later in life. However, the exact nature and time-course of these changes following injury, and the long-term effects of these changes on neuronal integrity, as well as the potential relationship between these and risk of PD development, remains unclear and requires further investigation.

### α-synuclein

In addition to imbalance in key neurotransmitter pathways, fibrillar aggregates of the α-synuclein protein in neuronal perikarya—in the form of Lewy Bodies—are a key pathological hallmark of PD [14, 148]. The first evidence of an association between α-synuclein and PD came from the discovery of rare familial forms of PD associated with mutations in the SNCA gene encoding α-synuclein [149, 150]. α-synuclein immunoreactivity and inclusions have since been observed in both sporadic and familial PD cases [151], with LBs extracted from the cortex, SN, hippocampus and striatum of individuals with PD shown to be composed primarily of the α-synuclein protein [152–155]. Furthermore, total [156, 157], oligomeric [158] and phosphorylated [159, 160] α-synuclein are observed at higher levels in the plasma of PwP than healthy controls, although reports are somewhat variable [160]. Increased levels of α-synuclein oligomers have also been observed in cerebrospinal fluid (CSF) of PwP and PD with dementia cases compared with controls [161, 162], although total protein levels are generally similar to [163, 164], or significantly lower than, control cases [165]. As such, both plasma and CSF α-synuclein have been suggested as potential biomarkers of disease [166]; however further evaluation of these as diagnostic markers in the prodromal phase of disease, and the role these changes play in disease development, is warranted [167].

#### Spread of α-Synuclein Pathology in PD

**Fig. 4:**
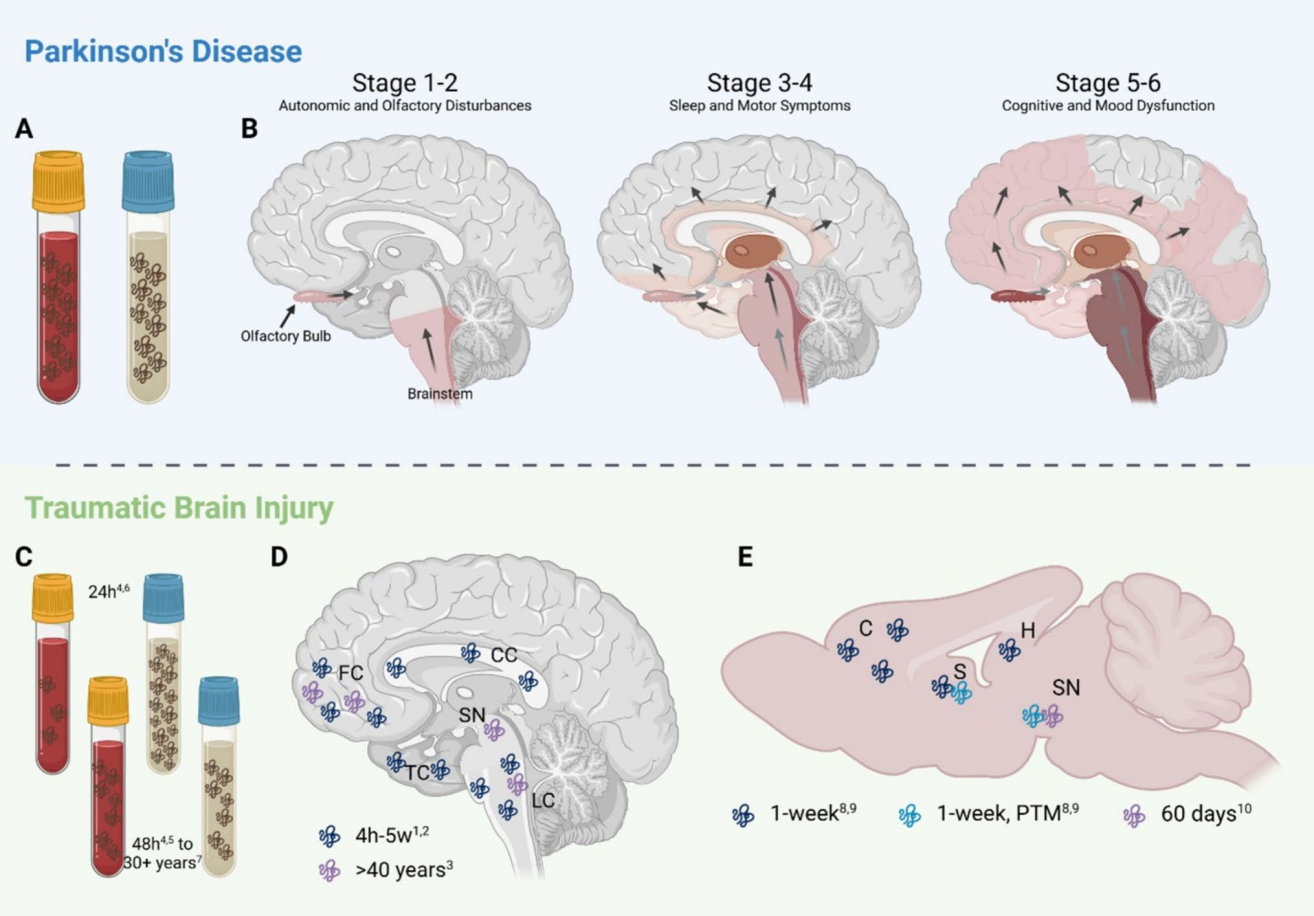
α-synuclein changes in PD and following injury. α-synuclein pathology spreads in a stereotypical pattern in PD (**B**; adapted from Braak et al., 2003 [14]), and has been identified in many of these affected sites in human post-mortem tissue following TBI (**D**) and preclinical models of injury (**E**). Additionally, oligomeric α-synuclein is increased in the plasma of PwP (**A**), while total protein levels are decreased acutely following injury, and return to normal by 48 h post-injury (**C**). In the CSF, oligomeric α-synuclein is increased in PwP (**A**), while protein levels are also increased acutely following injury but return to control levels by 48 h post-injury in adults (**C**). C: Cortex; CC: Corpus Callosum; FC: Frontal Cortex; H: Hippocampus; LC: Locus Coeruleus; S: Striatum; SN: Substantia Nigra; TC: Temporal Cortex. 1: Uryu et al., 2007; 2: Newell et al., 1999; 3: Crane et al., 2016; 4: Modello et al., 2013; 5: Ye et al., 2019; 6: Vorn et al., 2023; 7: Peltx et al., 2020, 8: Uryu et al., 2003; 9: Acosta et al., 2019; 10: Acosta et al., 2015. (Figure created with BioRender.com)

LB pathology spreads in a stereotypical pattern in most individuals with PD, comprehensively described by Braak et al. (2003) [14] in their model of disease staging (Fig. 4). Induction of pathology typically occurs in the dorsal motor nucleus and anterior olfactory nucleus and is associated with early presentation of non-motor symptoms, such as autonomic dysfunction and anosmia [14, 168, 169]. Spread of pathology from these regions is driven by their connectivity with other brain regions, and the susceptibility of these neuronal populations to degeneration [170]. Other symptoms, including motor and sleep disturbances, emerge as LBs spread to brainstem nuclei—such as the LC and SN—inducing cellular toxicity and degeneration in these areas [14, 104, 171]. As cortical involvement increases, PD patients experience more marked cognitive decline, as well as mood disturbance [14, 93]. In recent years, the gut-brain axis has also been proposed to play a critical role in the pathophysiology of PD and the spread of α-synuclein pathology (for review see Klann et al., 2022 [172]). In fact, the concept of “brain-first” vs “gut-first” subtypes of PD has been put forward, with those with so-called “gut-first” PD proposed to have pathological alpha-synuclein aggregates that originate in the enteric nervous system and are transmitted to the central nervous system via retrograde vagal transport (for review, see Borghammer & Van Den Berge, 2019 [173]). Alterations in the microbiota-gut-brain axis following TBI have also been proposed to play a role in neurodegenerative disease pathogenesis, although these investigations are in their infancy (for review, see Chiu and Anderson, 2023 [174]).

While the key pathological hallmark of PD is the presence of LBs in brain tissue, growing evidence suggests that LBs themselves are not cytotoxic, and instead play a protective role in isolating toxic misfolded α-synuclein species [175–179]. In particular, oligomeric protein species are thought to be the main source of cytotoxicity [180, 181], through interaction with and disruption of lipid membranes, and consequent cell death [181]. Evidence has demonstrated that α-synuclein oligomers and fibrils can be released into the extracellular space by exocytosis and can be endocytosed by both neuronal and non-neuronal cells, such as astrocytes and microglia [182, 183], where they seed misfolding of native healthy protein. As such, spread of α-synuclein throughout the brain may occur directly through neuronal connections, spreading α-synuclein pathology far beyond initial sites of misfolding and aggregation [182, 184]. Cell-to-cell transmission of α-synuclein has been demonstrated in vitro and in vivo [184, 185], in both rodent models of PD and human PD patients. Most notably, uptake of α-synuclein was observed in healthy human embryonic or foetal mesencephalic dopaminergic neuron grafts within 4 years of transplant into the striatum of people with PD [186, 187], and similar findings have since been reported in mouse models [182, 184]. Prolonged transmission of α-synuclein in this manner causes aggregation and LB pathology, with development of α-synuclein aggregations demonstrated in grafted cells within 16 years in individuals with PD, and 6 months in mouse models [182, 184–187]. Through these pathways, pathology has been observed to spread to the contralateral hemisphere and connected CNS regions, including brainstem nuclei, striatum, thalamus, hippocampus, occipital cortex, frontal cortex, and major white matter tracts, over the course of 15 months [171, 178, 179, 188]. The majority of spread occurs this way; however pathology may also be transmitted by non-neuronal cells or through the CSF [179].

#### Clinical Evidence of α-synuclein Pathology following TBI

Pathological loss of function and aggregation of α-synuclein has also been widely noted to occur after TBI. Following injury, axonal transport is disrupted, leading to accumulation of axonally transported materials, including α-synuclein, within the axon [189]. Early post-mortem studies of brain tissue from fatal TBI cases have demonstrated the presence of α-synuclein in axonal swellings in sections of the corpus callosum, frontal lobe, temporal lobe and brainstem between 4 h and 5 weeks post-injury [71, 190] (Fig. 4). Furthermore, Crane and colleagues identified a strong risk of Lewy Body pathology in TBI patients more than 40 years post-injury, with LOC < 1 h associated with LBs in the frontal and temporal cortex (RR = 1.64, 95% CI = 1.00–2.70), and LOC > 1 h associated with LBs in these regions and also the SN and LC (RR = 2.64, 95% CI = 1.40–4.99) [90]. These findings indicate that α-synuclein pathology occurs almost immediately after injury, but can also persist throughout one’s lifetime and develop in key regions associated with PD, potentially heightening the risk of PD development.

1: Uryu et al., 2007; 2: Newell et al., 1999; 3: Crane et al., 2016; 4: Modello et al., 2013; 5: Ye et al., 2019; 6: Vorn et al., 2023; 7: Peltx et al., 2020, 8: Uryu et al., 2003; 9: Acosta et al., 2019; 10: Acosta et al., 2015. (Figure created with BioRender.com).

Alterations to α-synuclein expression have also been noted in the CSF and blood of head trauma patients (Fig. 4). In line with this, levels of the protein were elevated within 24-h of injury in the CSF of adults who subsequently survived beyond 6-months following a severe TBI compared to controls, but returned to control levels by 48-h post-injury [191]. Similar changes have also been noted in severe paediatric TBI patients, with α-synuclein levels elevated in CSF approximately fivefold during the first 3 days post-injury, and rising to a > tenfold increase up to 6 days post-injury [192].

This is different to the pattern seen in blood-based studies of α-synuclein levels following injury. For example, a recent study of athletes with concussion found that α-synuclein was less abundant in the plasma in the first 48 h after injury [193]. A separate study of mild TBI inpatients found no significant difference in serum α-synuclein levels within a week of injury, but noted that lower serum α-synuclein levels were associated with more severe post-concussion symptoms and depression [194].

Thus, it appears that CSF and serum changes follow opposite trends in the acute phase post-injury, with increases in CSF levels and decreases in the serum. However, one-week post-injury is a broad time-frame, in which multiple fluctuations in protein levels may occur. Thus, further investigation of serum α-synuclein levels post-injury, in order to properly map the time-course of protein levels, is required in clinical populations in the acute phase following injury to better understand the changes occurring and how they may impact patient outcomes. Furthermore, these changes are in contrast to those seen in PwP, who generally display little change in CSF levels of α-synuclein [163], other than for oligomeric α-synuclein [161], and significant increases to α-synuclein levels (total, oligomeric and phosphorylated) in the blood [156, 158]. However, limited work has been conducted assessing chronic changes to CSF or serum and plasma levels of α-synuclein following TBI, with few studies beyond a week post-injury. One study of US veterans with history of TBI an average of 37 years prior found no differences in serum exosomal α-synuclein compared with shams, regardless of whether individuals were cognitively impaired or not [195]. Without further investigation of the time-course of changes throughout the chronic phase following injury, however, it is difficult to speculate on the nature of changes and what they may indicate about neuronal pathology and the development of neurodegeneration post-injury.

#### Preclinical Evidence of α-synuclein Pathology Following TBI

Preclinically, various models investigating the mechanisms behind injury-induced neurodegeneration have found significant changes in levels of α-synuclein in the brain following injury (Fig. 4). For example, early work by Uryu et al. observed a transient increase in α-synuclein immunoreactivity in the cortex, hippocampus and striatum at 1-week post-injury, and post-translationally modified forms of the protein in striatal axon bundles, that returned to baseline by 16 weeks, in a model of CCI that delivered impact over the left parietal lobe. Interestingly, this was isolated to mice injured at 24-months and was not seen in younger 4-month old mice, suggesting that age at the time of injury may impact a patient’s potential for recovery and, thus, the long-term outcomes they experience [196]. Utilising a similar CCI model, with impact delivered to the fronto-parietal cortex, Acosta et al. found comparable changes at 60 days post-injury in adult rats, reporting an increased number of α-synuclein -positive cells and an increased density of α-synuclein in dopaminergic cells of the SN ipsilateral to injury [114].

Furthermore, a negative correlation between the number of neurons with α-synuclein staining and the number of TH-positive cells in injured animals was noted following CCI injury, suggesting an association between α-synuclein accumulation and dopaminergic neurodegeneration following injury. Interestingly, this increase in α-synuclein was also positively correlated with the volume of inflammatory (MHC-II +) cells, indicating complex inter-relationships between α-synuclein upregulation, elevated inflammatory responses and dopaminergic loss that could contribute to the pathophysiology of PD development following injury [114]. In support of this, α-synuclein upregulation has been demonstrated to occur in both dopaminergic neurons and microglia in the midbrain 30 days after moderate CCI [120].

While these previous studies have focused on brain pathology following isolated injury events, effects of injury may also be cumulative, as demonstrated by Nogueira et al. investigating α-synuclein changes 10 days after prolonged (daily injury for 20 or 10 weeks) and more frequent injury (1, 2, 3, or 4 times per day for either study duration) exposure to varying severities of weight-drop injury. Significant main effects of impact energy, frequency and duration of exposure were observed on α-synuclein concentrations in whole brain tissue preparations, with the most severe, frequent and prolonged regimen (0.08 J impact energy, 4 × per day for 20 weeks) resulting in the highest levels of α-synuclein (a 30-fold increase compared to baseline), but even the least intense injury regimen (0.03 J impact energy, 1 × per day for 10 weeks) resulting in a fourfold change in α-synuclein levels [197]. Although minimally clinically and translationally relevant, due to the high frequency of repetitive injury, these findings provide insight into the progressive effects of head trauma on α-synuclein pathology.

It is important to acknowledge, however, that not all prior preclinical work has been consistent regarding α-synuclein levels post-injury. For example, while accumulation of α-synuclein was seen in SN neurons ipsilateral to injury in rats subjected to a combination of moderate LFP injury and prolonged pesticide exposure, no difference was found in α-synuclein immunoreactivity at either 11 days or 21 weeks post-injury in rats that received only the injury [69]. This discrepancy was also noted in another study by Acosta et al., which identified two different molecular weight proteins, both of which were confirmed to be α-synuclein**,** but which displayed differing responses in a model of mild blast injury. While the higher molecular weight (25 kDa) protein was increased in whole brain tissue preparations, as well as specifically in both the striatum and SN, at 2 and 7 days following blast TBI, the lower molecular weight (19 kDa) protein was decreased in the whole brain and striatum at both time points, but increased in the SN only at 7 days post-injury [118]. The authors speculated that the higher molecular weight protein may be a post-translationally modified form of α-synuclein, while the lower molecular weight protein is α-synuclein in its native form. This aligns with more recent work by Carlson et al., who found significantly lower concentrations of soluble monomeric α-synuclein in the cortex, hippocampus and striatum ipsilateral to CCI injury at various time-points post-injury, from 6 h up to 8 weeks [198]. However, further work elucidating the exact nature of the protein species being detected is required to better understand the conformational protein changes occurring following injury, in order to direct research and drug development toward the most significant and relevant of these targets.

To date, progressive spread of α-synuclein pathology throughout the brain from identified initiation sites, which is a key feature of PD [14], has not been characterised following TBI. This is in part due to the relative lack of TBI studies investigating the distribution of α-synuclein and related pathology in key initiation sites, such as the olfactory system and brainstem, and throughout the brain, both acutely and chronically (Table 2). However, the lack of α-synuclein pathology observed beyond a few months post-injury [69, 196] casts doubt over whether TBI could indeed play a role in initiating and perpetuating chronic α-synuclein accumulation, aggregation and spread, such as that seen in PD, or whether this is simply an acute response to injury that mimics some features of chronic neurodegenerative diseases. Given our current understanding of the importance of α-synuclein propagation in the development and progression of PD pathology, mapping the evolution of changes over time and throughout the brain—particularly in dopaminergic and noradrenergic pathways—following injury is critical for advancing our understanding of disease progression in TBI patients.

**Table 2:** Summary of a-synuclein changes following TBI

Region/tissue	Model	Outcome			Reference
		Acute	Sub-acute	Chronic	
**Whole brain**	Mild bast injury	↑25 kDa	↑25 kDa		Acosta et al., 2019
		↓19 kDa	↓19 kDa		
	Weight drop TBI (various severities and frequencies)		↑		Nogueira et al., 2018
**Cortex**	Mod. CCI (rats)	↓ m	↓ m	↓ m	Carlson et al., 2021
	CCI (mice)		↑	↔	Uryu et al., 2003
**Frontal lobe**	Human PM tissue	↗	↗		Uryu et al., 2007
				↑*	Crane et al., 2016
**Temporal lobe**		↗	↗		Uryu et al., 2007
				↑*	Crane et al., 2016
**Corpus callosum**			↗		Newell et al., 1999
**Internal capsule**			↗		
**Hippocampus**	Mod. CCI (rat)	↓ m	↓ m	↓ m	Carlson et al., 2021
	CCI (mouse)		↑	↔	Uryu et al., 2003
**Striatum**	Mod. CCI (rat)	↓ m	↓ m	↓ m	Carlson et al., 2021
	Mild bast injury	↑25 kDa	↑25 kDa		Acosta et al., 2019
		↓19 kDa	↓19 kDa		
	CCI (mouse)		↑ PTM		Uryu et al., 2003
**Brainstem**	Human PM tissue	↗	↗		Uryu et al., 2007
**Substantia nigra**	Human PM tissue			↑*	Crane et al., 2016
	Mild bast injury	↑25 kDa	↑19 kDa,25 kDa		Acosta et al., 2019
	Mod. LFP injury		↔	↔	Hutston et al., 2011
	mLFP + pesticide exposure		↑	↑	
	CCI (rat)			↑	Acosta et al., 2015
**Locus coeruleus**	Human PM tissue			↑*	Crane et al., 2016
**Midbrain**	Mod. CCI (mouse)		↑		Impellizzeri et al., 2016
**CSF**	Severe TBI patients (adult)	↑	↔		Mondello et al., 2013
	Severe TBI patients (paediatric)	↑	↑		Su et al., 2010
**Plasma/serum**	Athletes with concussion	↓			Vorn et al., 2023
	Mild TBI inpatients		↔		Ye et al., 2019
	TBI patients (US veterans)			↔	Peltz et al., 2020

CCI: controlled cortical impact injury; CSF: cerebrospinal fluid; LFP: lateral fluid percussion injury;

m = monomeric a-synuclein; PM: post-mortem; PTM: post-translationally modified forms

↗ a-syn present in axonal swellings

↑* increased LB risk

↔ no change compared to control or baseline

## The Role of Sex in the Link Between TBI and PD

Importantly, both TBI and PD occur more commonly in males [1, 38], and thus exploration of sex differences when considering the PD/TBI intersection is crucial, and often overlooked. Interestingly, while incidence is higher in males, TBI recovery tends to be worse in females [199] and disease progression follows differential patterns in males and females [200, 201]. However, findings regarding sex differences in outcomes following TBI are mixed, with significant discrepancy both within and between clinical and preclinical studies, as well as between different outcome measures – including functional and neuropathological changes – and different injury severities [202]. It has been suggested that hormonal and chromosomal differences, as well as related bioenergetic and biochemical features, may play a role in the sex differences observed (see Gupte et al. 2019 for review [202]), however further work is required to elucidate specific differences and the mechanisms driving these.

A limited number of studies have specifically assessed the effect of sex on the development of neurodegeneration following injury, with mixed results. In clinical populations, Crane et al. (2016) [90] noted no interaction between sex and TBI on neuropathological findings, including Braak Stage 5/6 and LBs in any region assessed. However, this study, and other epidemiological studies demonstrating a link between TBI and PD, did not specifically assess the interaction between sex and TBI on PD incidence or progression. In preclinical models, sex differences have been observed in the overall level of neurodegeneration, assessed by silver staining, following both diffuse and focal injury up to 7-days post-injury [203, 204], (although, of note, another study of CCI injury found no such differences using Fluoro-Jade C staining up to 7-days post-injury [205]). This difference was maintained out to 4-weeks following CCI injury [203], but no difference was observed by 14-days, and up to 60-days following diffuse injury [204]. Significantly, where sex differences were observed, the temporal profile of development and resolution of neurodegeneration differed between the sexes, with a later peak following CCI injury [204] and slower resolution in females following either injury [203]. Differences in immune responses at this acute time-point post-injury have also been noted, with significantly greater astrocyte reactivity and heme-oxygenase-1 (HO-1) staining (a marker of anti-oxidant molecules activated by various stressors) at 1-day post-injury in females [205]. In the sub-acute phase (14-days post-injury), sex differences were noted in the expression of a number of common genes used as biomarkers of injury, including glial fibrillary acidic protein (GFAP), enolase 2 (Eno2), microtubule-associated protein tau (MAPT) and brain-derived neurotrophic factor (BDNF) in the PFC and hippocampus following weight-drop or lateral impact injury [206]. Neuronal loss was not specifically investigated in this study, however these markers provide further insight into inflammatory processes, neuronal damage and neuronal growth and proliferation following injury, and are also commonly examined in the context of neurodegeneration. It should be noted, however, that pathology and degeneration of neurons during this acute/sub-acute phase likely reflects direct response to injury itself, and thus may not correlate well with long-term alterations in neuronal number, which could be indicative of increased risk of PD development.

However, longer-term studies assessing the interaction between sex and TBI at long-term time-points are limited. In a drosophila model of mild TBI, greater tissue degeneration was observed at 45-days post-injury in the cortex of females compared with males, although TUNEL (Terminal deoxynucleotidyl transferase dUTP nick end labelling) staining revealed limited signs of neuron death in either sex [207]. Transcriptomic profiles of injured animals differed between sexes both acutely (1 day) and chronically (6-weeks) post-injury, with 7 unique genes expressed in females at the chronic time-point, associated with immune and other (unidentified) functions [207]. While these findings provide early indications that sex differences in acute and chronic injury-related pathology may moderate the later risk of development of neurodegeneration, further work is required to elucidate the mechanisms via which this may occur. Importantly, while understanding of the role of TBI in the development of key pathological features of TBI – namely dopaminergic loss and α-synuclein aggregation and spread – is improving, particularly through the use of preclinical models of injury, the majority of these models use exclusively male cohorts, limiting our understanding of the impact of sex in these responses. Sex differences in chronic consequences of injury and concomitant underlying pathological mechanisms are particularly under-explored. Understanding the role of hormonal, immune and other biological disparities between the sexes will provide significant insight into this link, and improve the delivery of more equitable and effective early detection and intervention strategies.

## Conclusion

An overwhelming body of evidence exists indicating a link between TBI and the later development of PD. While this association has been acknowledged for decades, understanding of the biological mechanisms underlying this link is limited. Several parallels can be identified between the two pathologies, in both the biochemical and the neuronal changes observed. Of particular interest is the body of work indicating marked, although less significant in magnitude than that seen in PD, dopaminergic neuronal loss observed in the SN after injury, and changes in α-synuclein expression in PD-related regions, such as the SN and striatum.

Currently, the majority of research detailing pathological changes following TBI focusses on the acute phase, within hours and days of injury, and limited work has been done on changes beyond a few weeks post-injury. Indeed, these acute studies indicate that TBI can specifically impact key brain regions implicated in PD, such as the SN and striatum, and may therefore act as an important initiator of α-synuclein and other pathology, from which it can spread to connected regions and throughout the brain. However, with minimal preclinical work conducted to date beyond a few weeks post-injury, evidence of such spread of pathology following injury, and our understanding of the mechanisms underlying these changes and their longer-term effects, is limited.

Nevertheless, chronic reductions in dopaminergic signalling and the presence of α-synuclein and Lewy Body pathology from a limited number of clinical and preclinical cohorts indicate an induction of pre-neurodegenerative pathology following injury. Importantly, the role of neuroinflammation is likely key to understanding the link between TBI and PD diagnosis, with significant overlap between the neuroinflammatory upregulation observed in PwP and the inflammatory cascade known to be initiated following TBI. However, in order to fully understand the long-term ramifications of TBI for affected patients, focus on chronic outcomes, particularly relating to the development of neurodegenerative pathology, is needed. For patients who go on to develop PD years after TBI, this period presents an important opportunity for prevention and treatment of the progression and spread of pathology. Better understanding of the mechanisms occurring between TBI and PD diagnosis will allow the development of measures to identify individuals at risk of such complications, and potentially increase the efficacy of therapeutic interventions aimed at preventing this from occurring.

Importantly, while it is clear that pathological changes occur commonly post-injury, findings are somewhat contradictory, and evidence suggests that only a subset of individuals experience long-term effects from these changes. Thus, rather than TBI alone being the sole causative factor in the development of PD post-injury, investigation into the combined role of other factors, including genetics, lifestyle and other environmental exposures (e.g. pesticide use), may provide greater insight into the mechanisms at play and potential avenues for improved diagnosis and treatment of post-TBI sequelae and monitoring, diagnosis and treatment of PD development and progression.

## Data Availability

No datasets were generated or analysed during the current study.
